# Naturally derived cytokine peptides limit virus replication and severe disease during influenza A virus infection

**DOI:** 10.1002/cti2.1443

**Published:** 2023-03-23

**Authors:** Christopher M Harpur, Alison C West, Mélanie A Le Page, Maggie Lam, Christopher Hodges, Osezua Oseghale, Andrew J Gearing, Michelle D Tate

**Affiliations:** ^1^ Centre for Innate Immunity and Infectious Diseases Hudson Institute of Medical Research Clayton VIC Australia; ^2^ Department of Molecular and Translational Sciences Monash University Clayton VIC Australia; ^3^ Lateral Pharma Pty Ltd Melbourne VIC Australia

**Keywords:** cytokine, host‐targeted immunotherapy, influenza virus, pulmonary disease

## Abstract

**Objectives:**

Novel host‐targeted therapeutics could treat severe influenza A virus (IAV) infections, with reduced risk of drug resistance. LAT8881 is a synthetic form of the naturally occurring C‐terminal fragment of human growth hormone. Acting independently of the growth hormone receptor, it can reduce inflammation‐induced damage and promote tissue repair in an animal model of osteoarthritis. LAT8881 has been assessed in clinical trials for the treatment of obesity and neuropathy and has an excellent safety profile. We investigated the potential for LAT8881, its metabolite LAT9991F and LAT7771 derived from prolactin, a growth hormone structural homologue, to treat severe IAV infection.

**Methods:**

LAT8881, LAT9991F and LAT7771 were evaluated for their effects on cell viability and IAV replication *in vitro*, as well as their potential to limit disease in a preclinical mouse model of severe IAV infection.

**Results:**

*In vitro* LAT8881 treatment enhanced cell viability, particularly in the presence of cytotoxic stress, which was countered by siRNA inhibition of host lanthionine synthetase C‐like proteins. Daily intranasal treatment of mice with LAT8881 or LAT9991F, but not LAT7771, from day 1 postinfection significantly improved influenza disease resistance, which was associated with reduced infectious viral loads, reduced pro‐inflammatory cytokines and increased abundance of protective alveolar macrophages. LAT8881 treatment in combination with the antiviral oseltamivir phosphate led to more pronounced reduction in markers of disease severity than treatment with either compound alone.

**Conclusion:**

These studies provide the first evidence identifying LAT8881 and LAT9991F as novel host‐protective therapies that improve survival, limit viral replication, reduce local inflammation and curtail tissue damage during severe IAV infection. Evaluation of LAT8881 and LAT9991F in other infectious and inflammatory conditions of the airways is warranted.

## Graphical abstract

Novel host‐targeted therapeutics could treat severe influenza A virus infections, with reduced risk of drug resistance. Our studies provide the first evidence identifying LAT8881 and LAT9991F compounds, as novel host‐protective therapies that improve survival, limit viral replication and reduce local inflammation during severe influenza virus infection.

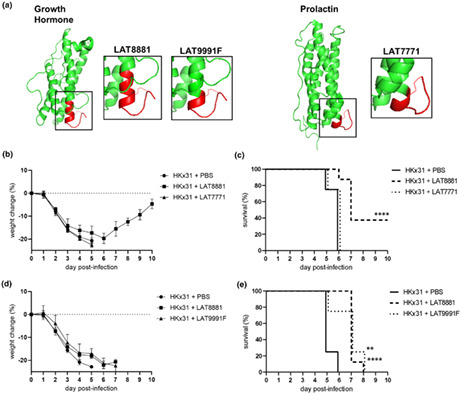

## Introduction

The coronavirus disease (COVID‐19) pandemic has demonstrated the global impact of the emergence of a novel respiratory virus in humans. There is also a constant threat that another influenza A virus (IAV) pandemic will occur. Therapeutic strategies for treating severe IAV infections have largely focussed on antivirals, which target viral proteins and have shown limited efficacy,^1^ because of the emergence of viral resistance and the need to administer them prophylactically or within the first 2 days of symptomatic infection.^2^ Severe IAV infections in humans are associated with hyperinflammation leading to the development of acute respiratory distress syndrome.^3^, ^4^ There is an urgent need to develop new host‐targeted immunotherapies that limit hyperinflammation‐driven morbidity and mortality, a common feature of severe respiratory virus infections.

Growth hormone (GH) is a member of the 4α‐helical cytokine family that regulates several biological processes acting via its cognate GH receptor.^5^ Growth hormone, like its structural homologue prolactin, is also known to be processed *in vivo* by several proteases at sites of tissue damage or pathology resulting in new active peptides. At least two major biologically active fragments of GH have been identified, a large fragment comprising the three N‐terminal α‐helices that has proposed anti‐angiogenic properties and a smaller C‐terminal fragment containing a di‐sulphide constrained loop.^6^, ^7^

LAT8881 is a synthetic form of the C‐terminal fragment of human GH, with the sequence YLRIVQCRSVEGSCGF, in which the two cysteines are linked by a disulphide bond.^8^, ^9^ Interestingly, LAT8881 (formerly identified as AOD9604) was found to not act via the GH receptor.^10^ Chronic oral and injected dosing of LAT8881 has been shown to reduce body weight, affect lipolysis and lipogenesis in fat tissue of obese rodents.^8^, ^10^, ^11^, ^12^ Intra‐articular injection has also been shown to reduce inflammatory damage in a rabbit collagenase model of osteoarthritis, resulting in an accelerated repair of the affected joint.^13^ Importantly, LAT8881 has been shown to have a promising profile in preclinical toxicology and in human safety studies.^14^, ^15^

The lanthionine synthetase C‐like protein (LANCL) family has recently been identified as putative targets for LAT8881.^16^ LANCL1 and the closely related family member LANCL2 are thought to be peptide‐modifying enzymes that act to protect cells and promote survival in the face of oxidative stress.^17^, ^18^, ^19^, ^20^, ^21^ Additionally, activation of LANCL2 by either natural or synthetic ligands reduced the severity of H3N2 and H1N1 IAV infection in mice, ameliorating inflammatory lung damage and accelerating recovery via immunoregulatory mechanisms.^22^, ^23^ Thus, based on its safety profile, tissue protective properties and potential association with LANCL protein signalling, we investigated the therapeutic effects of LAT8881 and related compounds in a preclinical model of severe IAV infection.

Here, we report that LAT8881 promotes cell viability and protects stressed cells from death *in vitro* via a mechanism that requires either LANCL1 or LANCL2. Critically, intranasal administration of LAT8881 or its metabolite, LAT9991F following IAV infection of mice, correlated with reduced viral loads and pro‐inflammatory cytokines in the lung, as well as evidence of reduced pulmonary injury. Moreover, LAT8881 treatment increased numbers of alveolar macrophages (AM), primary defenders of the airways and a key target for infection by IAV. In contrast, LAT7771 derived from the C‐terminus of the related prolactin protein had limited activity *in vivo*. Finally, we demonstrate markers of disease severity were further curtailed when LAT8881 was co‐administered with oseltamivir phosphate (OP), an approved influenza antiviral.

## Results

### LAT8881 promotes cell viability and protects against chemical‐induced death *in vitro*

**Figure 1:**
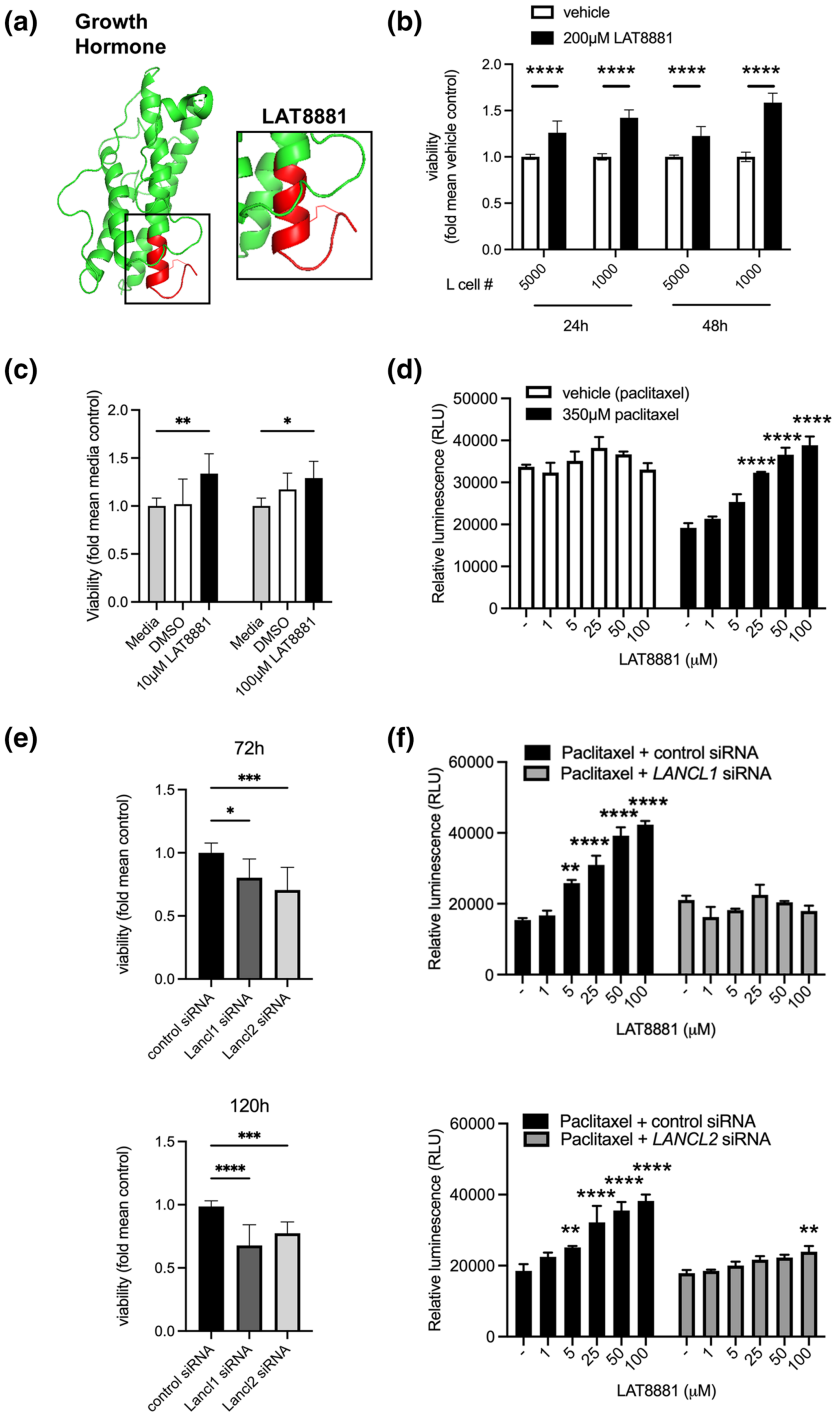
Human growth hormone‐derived LAT8881 improves *in vitro* cell survival in either a LANCL1 or LANCL2 protein‐dependent manner. **(a)** Schematic of human growth hormone structure (green, http://www.rcsb.org/structure/1HGU). The synthetic compound LAT8881 comprises the short C‐terminal region (red) and is cyclised by a disulphide bond between two cysteine residues as shown. **(b)** Viability of murine L cells (1000 or 5000 cells) relative to vehicle control samples following 24‐ or 48‐h incubation with LAT8881 (200 μM) or vehicle (DMSO) alone, as determined by luminescent ATP detection ± SD. ****P < 0.0001 vs vehicle control, Student's *t*‐test. Data are pooled from three independent experiments. **(c)** Viability of murine peritoneal exudate cells relative to media control in culture at 24 h following LAT8881 (10 μM) or vehicle (DMSO) alone, as determined by luminescent ATP detection ± SD. *P < 0.05, **P < 0.01 vs media control, two‐way ANOVA. Data are pooled from two independent experiments. **(d)** Viability of human A549 cells 16 h following 350 μM paclitaxel or vehicle (DMSO) alone ± LAT8881 (1–100 μM), as determined by luminescent ATP detection ± SD. ****P < 0.0001 vs paclitaxel without LAT8881, two‐way ANOVA. Data are representative of two independent experiments. **(e)** Viability of murine L cells transfected with 100 nM siRNA against *Lancl1* or *Lancl2* relative to control siRNA samples at 72 or 120 h, as determined by luminescent ATP detection ± SD. *P < 0.05, ***P < 0.001, ****P < 0.0001 vs control siRNA, one‐way ANOVA. Data are pooled from three independent experiments. **(f)** Viability of human A549 cells transfected with 100 nM siRNA specific to *LANCL1*, *LANCL2* or control siRNA 16 h following 350 μM paclitaxel ± LAT8881 (1–100 μM) or vehicle, as determined by luminescent ATP detection ± SD. **P < 0.001, ****P < 0.0001 vs paclitaxel + control siRNA without LAT8881, two‐way ANOVA. Data are representative of two independent experiments.

LAT8881 is a synthetic C‐terminal fragment derived from human GH (Figure 1a). To begin to elucidate a potential role for LAT8881 in mediating cell protection, we treated mouse fibroblast‐like L cells with 200 μM LAT8881 or vehicle alone (dimethyl sulfoxide (DMSO)). We observed that L cell viability was enhanced in the presence of LAT8881 following 24 and 48 h in culture relative to vehicle control (Figure 1b). Furthermore, 10 μM LAT8881 improved the viability of murine primary peritoneal exudate cells, suggesting its effects are not limited to nonmyeloid cells (Figure 1c). Interestingly, the prosurvival effect of LAT8881 was even more stark in the presence of cytotoxic stress, with human A549 alveolar epithelial cells displaying improved resistance to cell death induced by paclitaxel (Figure 1d) or hydrogen peroxide (H_2_O_2_; Supplementary figure 1) in a LAT8881 dose‐dependent manner. Recently, we identified that LAT8881 associates with LANCL1 in neuronal tissue from rats.^16^ Consistent with the reported role of LANCL proteins in mediating cell survival,^17^, ^19^, ^20^, ^21^ L cell viability markedly decreased 72 and 120 h following transfection with siRNA targeting *Lancl1* or *Lancl2* (Figure 1e). Finally, we observed that the ability of LAT8881 to improve the resistance of A549 cells to paclitaxel was lost in the presence of *LANCL1* or *LANCL2* siRNA (Figure 1f). Together, these data suggest LAT8881 has a prosurvival effect on various cell types *in vitro* via either LANCL1 or LANCL2 protein‐dependent pathways.

### Intranasal treatment with LAT8881 or LAT9991F promotes resistance to severe IAV infection

**Figure 2:**
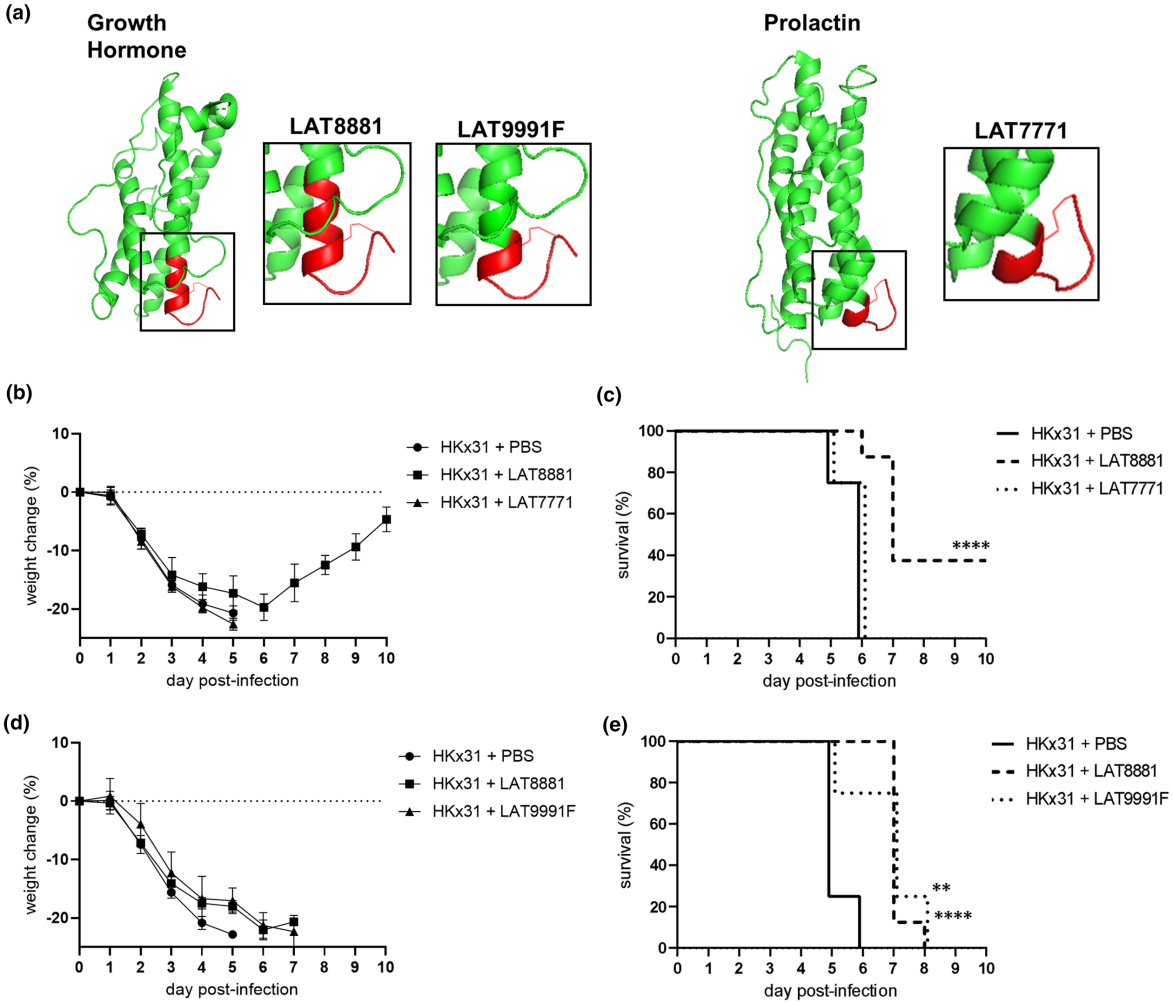
LAT8881 and its metabolite LAT9991F improves resistance to IAV infection *in vivo*. **(a)** Schematics of human growth hormone (GH; green, http://www.rcsb.org/structure/1HGU) and prolactin (green, http://www.rcsb.org/structure/1RW5) structures, which share a common four alpha helical bundle and a C‐terminal C‐C constrained loop. The synthetic compounds LAT8881, its metabolite LAT9991F from GH and LAT7771 from prolactin are cyclised peptides comprising short C‐terminal regions of the respective cytokines (red). Groups of male C57BL/6 mice (*n* = 8) received daily i.n. treatment with 20 mg kg^−1^ of LAT8881, PBS (vehicle control), 20 mg kg^−1^ LAT7771 **(b, c)** or 20 mg kg^−1^ LAT9991F **(d, e)** from 1 dpi with 10^4^ pfu of HKx31 IAV. **(b, d)** Mouse weight was recorded daily, and results are expressed as mean percent weight change ± SD. **(c)** Survival curves are shown. ****P < 0.0001 HKx31 vs HKx31 + LAT8881, Mantel–Cox log‐rank test. **(e)** Survival curves are shown. **P < 0.01 HKx31 vs HKx31 + LAT8881, ****P < 0.0001 HKx31 vs HKx31 + LAT9991F, Mantel‐Cox log‐rank test.

There is an urgent need to develop new therapeutics for respiratory viral infections, and an attractive approach is to target the host immune response, thus providing broad protection against multiple infectious agents without eliciting antiviral resistance. Development could be expedited by evaluating existing compounds that already have a demonstrated safety profile in humans. Therefore, we investigated the therapeutic effects of LAT8881 and related compounds in a preclinical mouse model of severe IAV infection. Male mice were infected with 10^4^ plaque‐forming units (pfu) of HKx31 (H3N2) IAV and subsequently received daily, intranasal (i.n.) treatments initiated 1‐day postinfection (dpi) with 20 mg kg^−1^ LAT8881 or its shorter metabolite LAT9991F (Figure 2a). Mice were also treated with 20 mg kg^−1^ of LAT7771, derived from the C‐terminus of prolactin, a cytokine hormone that is shares a common ancestral protein with GH (Figure 2a). Mice were monitored daily and euthanised either upon losing 20% of their initial body weight or displaying severe clinical signs of disease (see Methods). Treatment with LAT8881 reduced IAV infection‐induced weight loss, resulting in a significantly higher survival rate than mice that received LAT7771 or PBS vehicle (Figure 2b and c). Treatment with LAT9991F also prolonged the survival of mice, displaying similar efficacy to LAT8881 (Figure 2d and e). In contrast, LAT7771 derived from prolactin had no impact on weight loss or survival of mice. These data suggest that localised, therapeutic treatment with either of the two cyclised peptides derived from the C‐terminus of GH, LAT8881 and LAT9991F, can limit influenza disease symptoms.

### Intranasal treatment with LAT8881 or LAT9991F following IAV infection limits viral dissemination and inflammation

**Figure 3:**
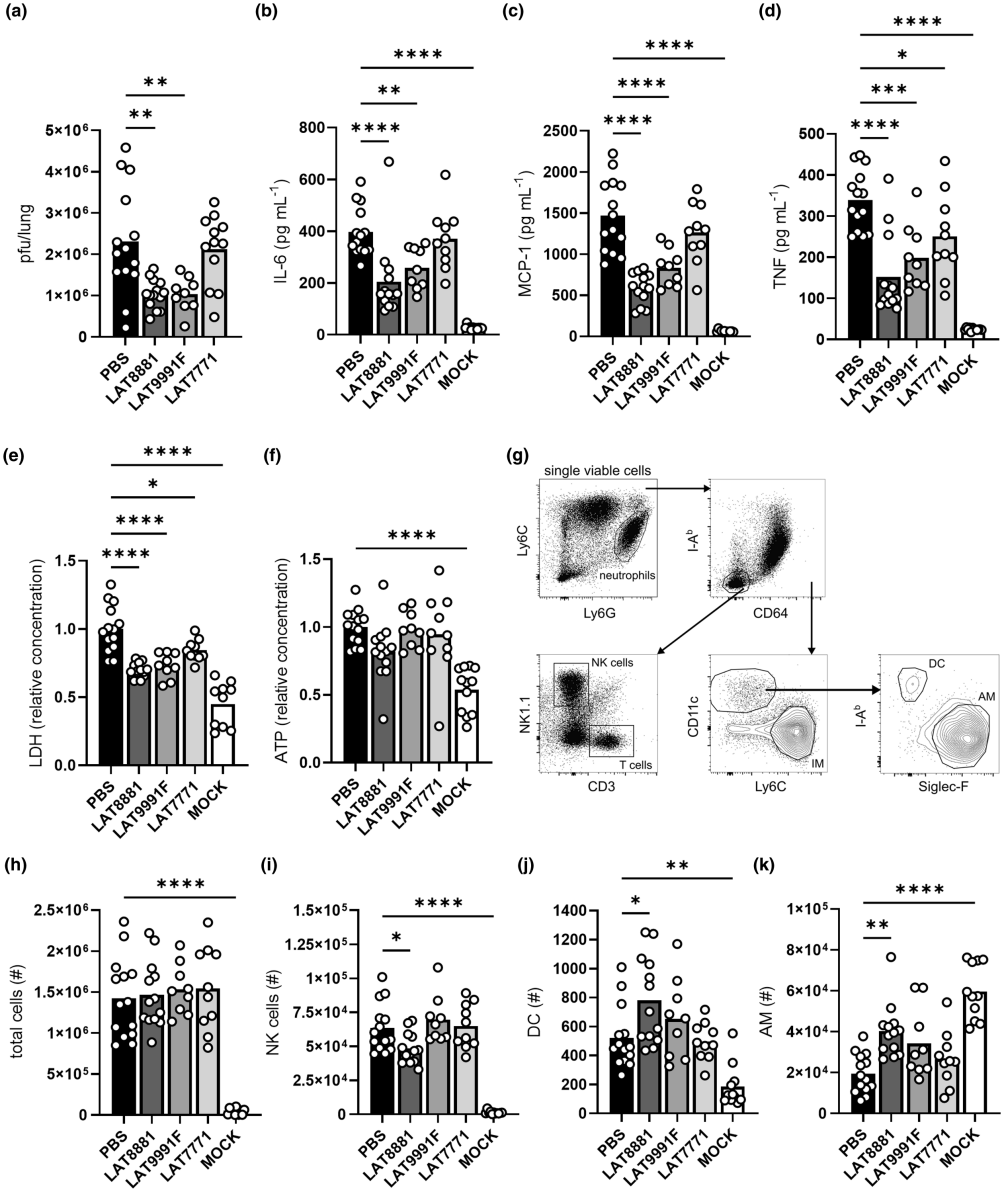
LAT8881 treatment during severe IAV infection reduces viral loads, inflammation and damage in the lung. Groups of male C57BL/6 mice received daily i.n. treatment with 20 mg kg^−1^ of LAT8881, LAT9991F or LAT7771 from 1 dpi with 10^4^ pfu of HKx31 IAV. BAL fluid and lung tissues were collected at 3 dpi. MOCK‐infected and IAV‐infected control mice received PBS alone. **(a)** Lung viral loads (pfu/lung) measured by a standard plaque assay. BAL fluid concentrations of IL‐6 **(b)**, MCP‐1 **(c)** and TNF **(d)** determined by cytokine bead array. Levels of LDH **(e)** and ATP **(f)** in BAL fluid relative to the PBS‐treated, IAV‐infected control mice determined by colorimetric and luminescent assays, respectively. **(g)** Representative flow cytometry gating strategy for BAL fluid immune cells. Numbers (#) of total viable cells **(h)**, NK cells **(i)**, DC **(j)** and AM **(k)** in the BAL fluid as determined by flow cytometry. Data are presented as the mean, pooled from at least two independent experiments, with each data point representing an individual animal. *n* = 9–14. *P < 0.05, **P < 0.01, ***P < 0.001, ****P < 0.0001, one‐way ANOVA with Dunnett's multiple comparisons test.

Host strategies to cope with influenza can involve either efficient clearance of the virus, or tolerance of the infection by reducing potential immunopathology and tissue damage.^24^ Severe IAV infections are characterised by excessive inflammation (also known as hyperinflammation) and cytokine storm, observed at both the site of infection and systemically. Therefore, we examined lung viral titres, as well as immune cells, damage markers and inflammatory cytokines in bronchoalveolar lavage (BAL) fluid in addition to circulating cytokine levels in male mice at 3 dpi with IAV (Figure 3). As per Figure 2, mice were infected with HKx31 IAV and subsequently received daily i.n. treatment with 20 mg kg^−1^ LAT8881, LAT9991F or LAT7771. Both IAV‐infected and MOCK‐infected control cohorts received i.n. PBS daily as a vehicle control.

Lung infectious viral burden (pfu/lung) was significantly reduced (> 2‐fold) 3 dpi by treatment with both LAT8881 and LAT9991F but not LAT7771 compared with infected PBS controls (Figure 3a). Interleukin‐6 (IL‐6), monocyte chemoattractant protein‐1 (MCP‐1) and tumor necrosis factor (TNF) are pro‐inflammatory cytokines commonly associated with influenza‐induced cytokine storms.^25^ Both LAT8881 and LAT9991F treatment regimens significantly reduced levels of IL‐6, MCP‐1 and TNF in BAL fluid, while only the concentration of TNF had slightly decreased with LAT7771 administration (Figure 3b–d). Of note, the concentrations of these key inflammatory mediators were still above their respective levels in the BAL fluid taken from MOCK‐infected control mice, which received PBS alone (MOCK). By contrast, no significant differences were observed in BAL fluid levels of interferon gamma (IFNγ), IL‐10 or IL‐12p70 (Supplementary figure 2a–c). Systemic levels of all assessed cytokines were also largely unchanged by treatment with LAT8881, LAT9991F or LAT7771, with only a slight, yet significant increase in circulating anti‐inflammatory IL‐10 observed in LAT8881‐treated mice compared with infected PBS controls (Supplementary figure 2d–i).

Extracellular lactate dehydrogenase (LDH) and adenosine triphosphate (ATP) are released by dead or dying cells, making them indicators of tissue damage. LAT8881 treatment was observed to significantly reduce LDH in BAL fluid relative to infected PBS controls (Figure 3e), with levels of the shorter‐lived danger‐associated molecular pattern (DAMP) ATP also trending lower (Figure 3f). LAT9991F also significantly reduced LDH in the BAL fluid but had a more negligible effect on ATP. As inflammation and tissue damage appeared to diminish with these treatments, we investigated whether there had also been any changes in the innate immune cell composition of the airways. Accordingly, we enumerated total cells, neutrophils, natural killer (NK) cells, T cells, inflammatory macrophages (IM), dendritic cells (DC) and AM within the BAL fluid at 3 dpi using flow cytometry (Figure 3g). All IAV‐infected mice had comparable total airway cellularity (Figure 3h), as well as similar numbers of T cells, IM and neutrophils (Supplementary figure 3a–c). As expected, the BAL fluid of MOCK controls that subsequently received daily i.n. treatment with PBS had relatively few cells (Figure 3h). Only LAT8881 treatment induced a significant decrease in airway NK cells and increase in DC numbers 3 dpi (Figure 3i and j).

Resident in the lumen of the alveoli, AM are susceptible to infection by HKx31 IAV and play an important protective role in limiting lung viral loads.^26^, ^27^, ^28^ Both i.n. administration of 20 mg kg^−1^ LAT8881 and LAT9991F resulted in greater numbers of AM in the BAL fluid, compared with infected mice that received i.n. PBS alone, yet the increase was only statistically significant in mice treated with LAT8881 (Figure 3k). Of note, while i.n. LAT8881 treatment almost doubled AM abundance in the BAL of IAV‐infected mice, the number of AM was still fewer than in MOCK controls. Therapeutic LAT8881 and LAT9991F treatment may therefore maintain AM *in vivo* potentially by protecting them against cell death. Epithelial cells lining the airways are the major cell type that supports IAV replication. Having established that i.n. LAT8881 administration limits IAV loads *in vivo*, we examined the ability of LAT8881 to limit viral replication in human primary bronchial epithelial cells (PBECs). Indeed, the addition of 100 μM LAT8881 1 h after infection of PBECs with HKx31 IAV resulted in decreased levels of infectious virus in cell culture supernatants at 24 h (Supplementary figure 4a). PBECs treated with LAT8881 were more viable than those treated with the vehicle control, suggesting the decreased level of infectious virus was not because of increased cell death (Supplementary figure 4b). Collectively, these data suggest that the increase in influenza disease resistance provided by local administration of LAT8881 and to a lesser degree, LAT9991F, correlates with decreases in tissue damage, inflammation and IAV titres in the lung, as well as the retention of greater numbers of AM, a key mediator of early defence against infection. Furthermore, our *in vitro* results suggest that LAT8881 can limit viral replication in epithelial cells, which may also influence the amount of AM we observe in the airways during IAV infection of mice.

### Dose‐dependent modulation of virus dissemination and the innate immune response to IAV infection by LAT8881

**Figure 4:**
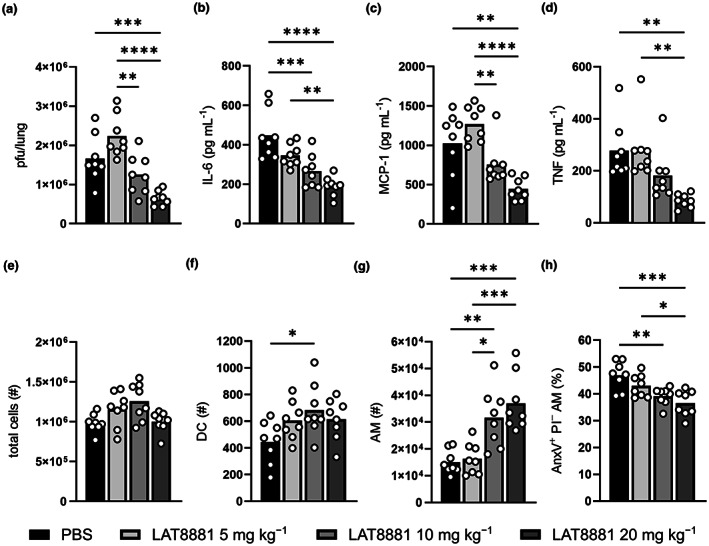
Effectiveness of LAT8881 treatment of severe IAV infection is dose‐dependent. Groups of male C57BL/6 mice received daily i.n. treatment with 5, 10 or 20 mg kg^−1^ LAT8881 from 1 dpi with 10^4^ pfu of HKx31 IAV. BAL fluid and lung tissues were collected at 3 dpi. IAV‐infected control mice received PBS alone. **(a)** Lung viral loads (pfu/lung) were measured by a standard plaque assay. BAL fluid concentration of IL‐6 **(b)**, MCP‐1 **(c)** and TNF **(d)** determined by cytokine bead array. Numbers (#) of total viable cells **(e)**, DC **(f)** and AM **(g)** in the BAL fluid as determined by flow cytometry. **(h)** Percentage of Annexin V^+^ PI^−^ (AnxV^+^ PI^−^) AM of total AM cells in the BAL fluid. Data are presented as the mean from a single experiment, with each data point representing an individual animal. *n* = 8 per group. *P < 0.05, **P < 0.01, ***P < 0.001, ****P < 0.0001, one‐way ANOVA with Tukey's multiple comparisons test.

To confirm that the observed changes in IAV‐infected mice treated with 20 mg kg^−1^ LAT8881 were indeed the result of the localised administration of the compound, we examined the effect of different doses of LAT8881 on lung infectious viral loads, as well as the inflammatory cytokines and immune cells within the BAL fluid. Male mice were infected with HKx31 IAV and administered 5, 10 or 20 mg kg^−1^ LAT8881 i.n. each day from 1 dpi. An additional IAV‐infected control cohort received i.n. PBS daily as a vehicle control. The reduction in viral burden in the lung and the concentration of IL‐6, MCP‐1 and TNF in the airways inversely correlated with increasing dose of LAT8881 (Figure 4a–d). The effects were most marked with the two highest doses, although only IL‐6 was reduced to a significant degree by 10 mg kg^−1^ LAT8881 compared with treatment with PBS. Total cell numbers in the BAL fluid were not significantly affected by any treatment, while both the amount of DC and AM trended higher with increasing LAT8881 dose (Figure 4e–g). Congruent with this, the frequency of dying (Annexin V^+^ PI^−^) AM decreased with increased LAT8881 dose (Figure 4h). Importantly, analysis of multiple readouts of disease severity in IAV‐infected female mice treated with 20 mg kg^−1^ LAT8881 (Supplementary figure 5) revealed similar trends to that of male mice (Figures 3 and 4).

### LAT8881 reduces pulmonary pathology during severe IAV infection

**Figure 5:**
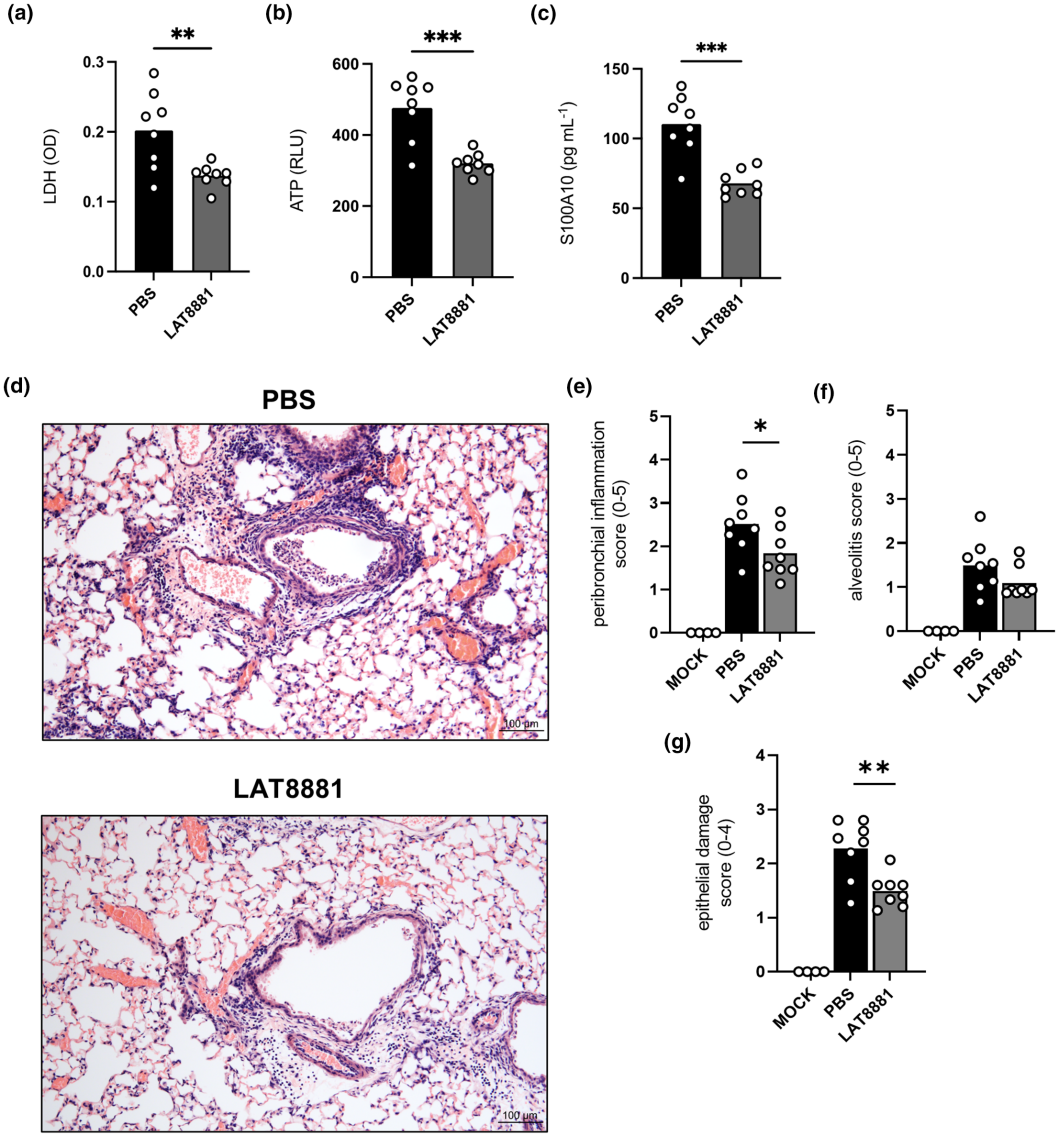
LAT8881 treatment of severe IAV infection reduces pulmonary immunopathology. Groups of male C57BL/6 mice received daily i.n. treatment with 20 mg kg^−1^ of LAT8881 or PBS alone from 1 dpi with 10^4^ pfu of HKx31 IAV. BAL fluid was collected at 3 dpi. Levels of LDH **(a)**, ATP **(b)** and S100A10 **(c)** in BAL fluid determined by colorimetric (OD; optical density), luminescent (RLU; raw luminescence units) assays or ELISA. Data are presented as the mean with each data point representing an individual animal. *n* = 8 per group from one experiment. **P < 0.005, ***P < 0.001, Student's *t*‐test. Groups of male and female C57BL/6 mice received daily i.n. treatment with 20 mg kg^−1^ of LAT8881 or PBS alone from 1 dpi with 10^4^ pfu of HKx31 IAV. Lungs underwent formalin fixation at 3 dpi and histological analysis of H&E‐stained lung tissue sections were performed. **(d)** Representative images at 10× magnification (scale bar = 100 μm). Lung sections were randomised and scored blind by 3 readers for **(e)** peribronchial inflammation (scale 0–5), **(f)** alveolitis (scale 0–5) and **(g)** epithelial damage (scale 0–4), as described in the Methods. Data are presented as the mean with each data point representing an individual animal. *n* = 4–8 per group from one experiment. *P < 0.05, **P < 0.01, only PBS vs LAT8881 shown, one‐way ANOVA with Dunnett's multiple comparisons test.

Having established that 20 mg kg^−1^ LAT8881 limits LDH levels in BAL fluid (Figure 3e, Supplementary figure 5f), suggestive of reduced lung tissue damage, we performed further analysis on an additional cohort of animals. At 3 dpi, the marked reductions in LDH and ATP levels in BAL fluid were reproduced following 20 mg kg^−1^ LAT8881 treatment of IAV‐infected mice (Figure 5a and b). Furthermore, the concentration of another DAMP molecule, S100 calcium‐binding protein A10 (S100A10) significantly decreased in BAL fluid with LAT8881 administration (Figure 5c). Interestingly, histopathological analysis of lung tissue sections (Figure 5d) indicated that peribronchial inflammation was significantly diminished in LAT8881‐treated mice at 3 dpi, while a more moderate reduction in alveolitis was observed (Figure 5e and f). Finally, treatment with LAT8881 significantly reduced epithelial damage compared with infected PBS controls (Figure 5g). Together, these data demonstrate LAT8881 treatment curtails IAV‐induced pulmonary tissue damage.

### Commencement of LAT8881 treatment at the onset of severe influenza disease reduces inflammation in the airways

As patients often only present to hospital with severe influenza disease pathology, we examined the impact of commencing LAT8881 closer to the previously observed onset of severe disease in our IAV infection model (Figure 2).^29^ Therefore, HKx31 IAV‐infected male mice were administered with either 20 mg kg^−1^ LAT8881 or PBS vehicle control i.n. on 3 and 4 dpi rather than 1 dpi. At 5 dpi, lung viral loads were markedly lower than at 3 dpi (Figure 3a), as the infection started to resolve.^28^ Indeed, infectious virus titres at 5 dpi were less than half of what we observed in infected PBS control mice at 3 dpi, and while lung viral burden in LAT8881‐treated mice trended lower, this was not significantly different to PBS controls (Supplementary figure 6a). LAT8881 had negligible effects on the concentration of IL‐6 in BAL fluid but did induce reductions in the concentrations of MCP‐1 and TNF, significantly so for the latter (Supplementary figure 6b–d). Modest reductions in the levels of ATP and LDH in BAL fluid were also observed following LAT8881 treatment (Supplementary figure 6e and f). Total cell numbers in the BAL fluid were comparable with PBS controls following LAT8881 treatment; however, NK cell and T cell numbers were significantly reduced (Supplementary figure 6g–i). Finally, AM abundance was significantly elevated in LAT8881‐treated mice (Supplementary figure 6j) despite the more delayed treatment, which is consistent with results obtained when treatment began 1 dpi (Figures 3k and 4g). Collectively, these data suggest local administration of LAT8881 following the development of severe influenza disease still limits inflammation and maintains the critical AM population in the airways.

### LAT8881 treatment has a comparable therapeutic efficacy to the IAV antiviral oseltamivir phosphate

Having established that intranasal treatment with LAT8881 limits viral spread and potentially deleterious inflammation, we compared its therapeutic efficacy against the existing prescription antiviral oseltamivir phosphate (OP), which inhibits the influenza neuraminidase (NA) protein.^30^ Although the clinical effectiveness of OP in reducing mortality is in question,^1^ particularly in the context of the emergence of OP‐resistant strains of influenza,^31^ oral administration of OP has been shown to limit IAV disease in animal models using controlled infection parameters.^23^, ^32^, ^33^ As such, we evaluated therapeutic daily i.n. treatment with 20 mg kg^−1^ LAT8881 or PBS vehicle, as well as oral gavage delivery of 10 mg kg^−1^ OP or PBS vehicle in our model of IAV infection. We additionally co‐administered LAT8881 and OP to look for any potential additive therapeutic benefit. As before, infected mice treated with PBS alone were used as a vehicle control cohort.

**Figure 6:**
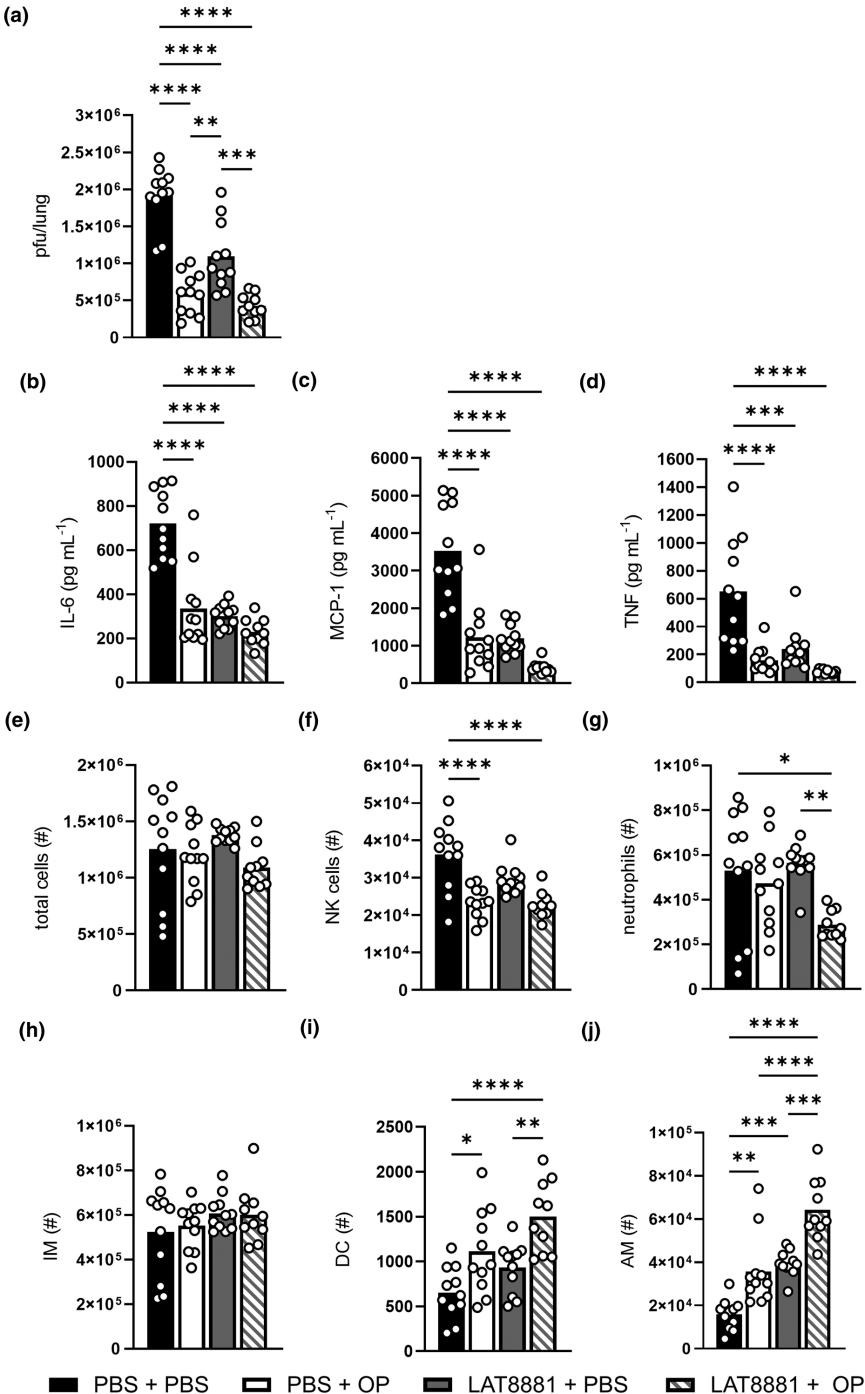
LAT8881 treatment of severe IAV infection has similar efficacy to antiviral oseltamivir phosphate. Groups of male C57BL/6 mice received daily i.n. treatment with either PBS or 20 mg kg^−1^ LAT8881 concurrent with either PBS or 10 mg kg^−1^ oseltamivir phosphate (OP) via oral gavage from 1 dpi with 10^4^ pfu of HKx31 IAV. Mice were sacrificed on 3 dpi and BAL fluid and lung tissues were collected. **(a)** Lung viral loads (pfu/lung) were measured by a standard plaque assay. BAL fluid concentration of IL‐6 **(b)**, MCP‐1 **(c)** and TNF **(d)** determined by cytokine bead array. Numbers (#) of total viable cells **(e)**, NK cells **(f)**, neutrophils **(g)**, inflammatory macrophages (IM) **(h)**, DC **(i)** and AM **(j)** in the BAL fluid as determined by flow cytometry. Data are presented as the mean, pooled from two independent experiments, with each data point representing an individual animal. *n* = 10–11 per group. *P < 0.05, **P < 0.01, ***P < 0.001, ****P < 0.0001, one‐way ANOVA with Tukey's multiple comparisons test.

Interestingly, we observed a significant decrease in lung viral titres at 3 dpi in mice that received OP alone commensurate with its ability to limit IAV replication (Figure 6a). LAT8881 + PBS treatment alone almost halved the amount of infectious virus in the lung, when compared to mice that received PBS alone. Importantly, co‐treatment with LAT8881 + OP reduced lung viral titres to a similar or even lower level than PBS + OP or LAT8881 + PBS. An analogous pattern was observed for concentrations of IL‐6, MCP‐1 and TNF in BAL fluid at 3 dpi, with PBS + OP and LAT8881 + PBS treatment regimens inducing similar decreases in these pro‐inflammatory cytokines compared with PBS + PBS control mice, with LAT8881 + OP co‐treatment promoting even greater reductions (Figure 6b–d).

Consistent with our previous results, total cellularity in the airways in IAV‐infected mice was similar regardless of treatment (Figure 6e). NK cell abundance had decreased with all experimental treatment regimens; however, this was most pronounced in treatments including OP (Figure 6f). Interestingly, while LAT8881 + PBS or PBS + OP had minor effects on airway neutrophil numbers, mice that received LAT8881 + OP had significantly fewer neutrophils than the PBS + PBS control cohort (Figure 6g). This decrease in neutrophils may reflect a further dampening of the inflammatory milieu and diminished viral burden. The infiltration of IM was seemingly unaffected by any experimental treatment regimen (Figure 6h). Finally, mice treated with LAT8881 or OP individually or in combination had significantly elevated DC and AM numbers, although the increase was most notable in mice that received LAT8881 + OP (Figure 6i and j). In sum, these data suggest i.n. LAT8881 has similar efficacy as a therapeutic treatment for severe IAV infection, to an antiviral medication already in use in the clinic. Additionally, when used in combination, LAT8881 and OP had improved effects on several established correlates of disease severity, chiefly decreased pro‐inflammatory cytokines levels, a reduction in infiltrating neutrophils and an increase in resident AM numbers.

## Discussion

Severe and fatal IAV infections are associated with hyperinflammation, cytokine storm and tissue damage. Without novel host‐targeted therapeutics to limit the development of severe disease, we are ill‐prepared for an inevitable IAV pandemic. Identifying existing host‐targeted compounds that help defend against these viral pathogens and/or ameliorate disease symptoms is a high priority, as they could be more rapidly introduced into the clinic, thereby reducing the healthcare and economic burden, while limiting the potential drug resistance associated with antiviral medication.

LAT8881 is a synthetic version of the C‐terminal fragment of GH, the activity of which is not dependent on a functional interaction with the GH receptor.^8^, ^9^, ^10^, ^11^ LAT8881 can undergo further N‐terminal cleavage *in vivo* to a peptide CRSVEGSCGF (LAT9991F) that retains its disulphide bond and ostensibly its cyclic 3D structure.^9^, ^34^, ^35^ Proteolytic processing of GH at sites of tissue damage and inflammation (e.g. in viral lung infection or osteoarthritis) to release C‐terminal fragments may represent an endogenous pathway to help limit inflammatory damage and promote recovery. Furthermore, LAT8881 does not have any of the systemic hormonal effects of GH, including no changes in insulin‐like growth factor 1 production, which may exacerbate IAV‐induced lung damage and increase disease susceptibility.^14^, ^36^

We demonstrated that LAT8881 promotes the viability of a range of cell types *in vitro* including in the presence of inducers of cytotoxic stress (Figure 1). Additionally, LAT8881 treatment of IAV‐infected mice reduced the proportion of AMs displaying the early cell death marker Annexin V in a dose‐dependent manner (Figure 4h). Together these data suggest a role for LAT8881 in stimulating cellular pathways responsible for survival particularly under various conditions of stress. We recently observed that LAT8881 interacts with the LANCL1 in rat neurons.^16^ In line with this, we found siRNA targeting of *LANCL1* or *LANCL2* limited cell viability (Figure 1e) and abolished the LAT8881 effects (Figure 1f, Supplementary figure 1). The ability of LANCL proteins to promote cell survival is consistent with the literature.^17^, ^19^, ^20^, ^21^ In direct comparison with our data, Wang *et al*. demonstrated that while overexpression of LANCL1 in human prostate cancer cell lines enhanced cell viability and conferred resistance to cell death induced by hydrogen peroxide, LANCL1 knockdown sensitised cells to oxidative stress.^20^ The involvement of LANCL2 in cell survival has been associated with Akt signalling, whereby *LANCL2* knockdown in HepG2 liver cells leads to increased rates of apoptosis.^21^ Along with roles in promoting cell survival, the LANCL1 and LANCL2 proteins have proposed immunomodulatory functions. Tan *et al*. demonstrated central nervous system‐specific deletion of LANCL1 heightened mRNA levels of *TNF* and *IL6*, alongside increased neuronal apoptosis and neurodegeneration.^19^

Recovery from infection can result from efficient virus elimination or by tolerance of the infection via limiting the associated immunopathology.^24^ Influenza disease outcomes may therefore be dictated by pathogen virulence and host resistance to and/or tolerance of the infection. Crucial to this is striking a balance between swift virus elimination and regulation of the immune response and potential hyperinflammation. In our present study, therapeutic treatment with LAT8881 or its metabolite LAT9991F successfully reduced lung viral replication and pro‐inflammatory mediators during the acute phase of infection, which correlated with lessened disease symptoms and significantly improved survival (Figure 2). We see significant rapid reductions, but importantly not total depletion, of pro‐inflammatory cytokines IL‐6, TNF and MCP‐1 in the airways (Figure 3b–d) elicited by treatment with LAT8881 and LAT9991F. This may have tuned the local innate immune response to better counteract the infection, while also limiting excessive tissue damage that can exacerbate inflammation, as evidenced by reduced levels of LDH and the DAMP molecule S100A10 in BAL fluid (Figures 3e and 5a–c). Indeed, histological analysis of lung tissue sections from LAT8881‐treated mice revealed reduced peribronchial inflammation and features associated with epithelial damage (Figure 5d–g).

In the context of influenza, LANCL2‐deficient mice have higher levels of IL‐6 and MCP‐1 in their lungs, while the oral treatment of infected mice with selective LANCL2 ligands abscisic acid and NSC61610 reduced the levels of TNF and MCP‐1 in the lung during IAV infection.^22^, ^23^ The anti‐inflammatory effect of LAT8881 and LAT9991F during IAV infection, therefore, is consistent with these previous reports of LANCL2‐targeted treatment *in vivo*; however, in contrast to those studies, LAT8881 and LAT9991F treatment also decreased viral titres in the lung (Figures 3a and 4a). Indeed, a reduction in IAV propagation was observed *in vitro* in primary human bronchial epithelial cells (PBECs) treated with LAT8881 (Supplementary figure 4a), suggesting LAT8881 may act on epithelial cells lining the airways to limit IAV replication. In this experiment, LAT8881 was added 1 h following IAV infection, indicating the observed reduction in viral replication was not because of direct interaction with the virus itself. However, a limitation with this approach is that the PBECs were not grown in an air liquid interface and therefore more closely emulate basal cells rather than epithelial cells of the bronchus.

Alveolar macrophages are the sentinels of the airways and a primary target of IAV infection which results in cell death without release of infectious virus,^26^, ^27^, ^28^ and loss of AM has also been observed in hospitalised patients with moderate and severe COVID‐19.^37^ HKx31 IAV infection of mice reported here resulted in a rapid reduction of AM in the airways of PBS‐treated controls (Figure 3k). Whereas the loss of AM following IAV infection of LANCL2‐deficient mice is even more profound than in wildtype controls,^23^ LAT8881 and LAT9991F treatment was associated with greater numbers of AM in the airways (Figures 3k and 4g), suggesting both compounds may limit their IAV infection‐induced depletion. Significantly, delaying commencement of LAT8881 treatment until 3 dpi, the onset of severe disease, also resulted in greater numbers of AM (Supplementary figure 6j). Indeed, it appears that LAT8881 treatment may promote AM longevity during IAV infection as the proportion of AM displaying the early cell death marker Annexin V, decreased with increased dose of LAT8881 (Figure 4h). Pneumonia is a frequent influenza complication as either a direct consequence of IAV infection or, more commonly, secondary bacterial infections.^38^, ^39^ As such, LAT8881‐ and LAT9991F‐mediated reductions in lung viral burden, modulation of inflammation in the airway microenvironment and maintenance of a denser population of AM at the site of infection, could help protect against additional viral infections, as well as the development of viral or bacterial pneumonia by promoting quicker resolution of primary virus infections via effective and proportionate immune responses.^27^, ^40^, ^41^

Influenza A virus infection triggers a rapid influx of leukocytes from the circulation into the lung, dominated by IM and neutrophils and sustained by their release of chemokines.^29^, ^42^ Interestingly, we observed no major changes in these key infiltrating leukocytes (Supplementary figure 3) with LAT8881 or LAT9991F treatment alone; however, neutrophils have significant cytotoxic potential and dysregulated neutrophil activation can contribute to lung injury and lethal disease.^43^, ^44^, ^45^ Specific antibody‐mediated depletion of neutrophils reportedly results in worse survival, weight loss and increased extrapulmonary spread in mouse models of IAV infection.^46^, ^47^, ^48^ Meanwhile, antibody‐mediated reduction, but not complete depletion, of neutrophils during infection with PR8 H1N1 IAV improved disease.^43^ This suggested that a measured reduction in lung infiltrating neutrophils could limit neutrophil‐associated immunopathology while retaining their contribution to early host defence against the virus. In line with this, significantly fewer neutrophils were observed in the BAL fluid from IAV‐infected mice co‐treated with LAT8881 and OP, which was greater than that elicited by each individual treatment where neutrophil abundance was unchanged compared with PBS controls (Figure 6g). Whether this reflected reduced neutrophil infiltration or longevity has not been established yet; however, this reduction correlated with a further reduction in IL‐6 (Figure 6b), a cytokine that can protect neutrophils from influenza‐induced cell death.^46^

In sum, we report that LAT8881 and its truncated metabolite LAT9991F provided a therapeutic benefit in a preclinical IAV infection model, while LAT7771, derived from a homologous C‐terminal region of prolactin, had negligible effects. This suggests that the protective properties of LAT8881 and LAT9991F are not shared by structurally similar peptides from one of growth hormone's close relatives, nor by cyclic peptides in general. Based on its established safety profile in animals and humans,^14^, ^15^ LAT8881 shows promise as a potential treatment for severe IAV infection, and we also intend to extend our future investigations of this compound, and the derivatives thereof, to alternate inflammatory lung diseases including asthma, chronic obstructive pulmonary disease (COPD) and COVID‐19.

## Methods

### Compound generation

LAT8881 is a 16‐amino‐acid synthetic form of the C‐terminal fragment of human GH (H‐YLRIVQCRSVEGSCGF‐OH), which contains an additional N‐terminal tyrosine residue and two cysteine residues linked by a disulphide bond. LAT9991F is a 10‐amino‐acid synthetic peptide (H‐CRSVEGSCGF‐OH), which is a truncated form and a known metabolite of LAT8881.^34^ LAT7771 (H‐CRIIHNNNC‐OH) is the 9‐amino‐acid structural homologue of LAT9991F, derived from prolactin and was included as a control peptide. The cysteines in both LAT9991F and LAT7771 were also disulphide linked. All peptides were synthesised by Auspep Pty Ltd (Melbourne, Australia).

### *In vitro* treatment of cells with compounds

The mouse fibroblast L cell line (RRID:CVCL_8887) was grown in RPMI (Thermo Fisher Scientific, Waltham, USA, Cat #11875119) supplemented with 10% (v/v) heat‐inactivated FBS and 2 mM L‐glutamine (Thermo Fisher Scientific, Waltham, USA, Cat #25030–081) and seeded into 96‐well plates, at the indicated densities. Peritoneal exudate cells (PECs) were obtained from untreated C57BL/6J mice by flushing the peritoneal cavity with 5 mL PBS and adherence to 96‐well plates overnight in DMEM (Thermo Fisher Scientific, Waltham, USA, Cat #11965118) supplemented with 10% (v/v) heat‐inactivated FBS, 1% (v/v) penicillin/streptomycin (Thermo Fisher Scientific, Waltham, USA, Cat #15140122) and 2 mM L‐glutamine (Thermo Fisher Scientific, Waltham, USA, Cat #25030–081), at the indicated densities. Triplicate wells of L cells and PECs were treated with LAT8881 (10–200 μM as indicated) or an equivalent volume of DMSO vehicle. In some experiments, L cells were transfected with 100 nM siRNA specific for mouse *Lancl1* (Ambion *In Vivo*, Life Technologies, Carlsbad, USA, Cat #4457308), *Lancl2* (Ambion *In Vivo*, Life Technologies, Carlsbad, USA, Cat #4457308) or control siRNA (Ambion *In Vivo* Negative Control #1; Cat #4457289) using Lipofectamine 3000 (Thermo Fisher Scientific, Waltham, USA, Cat #L3000015). Cell viability was assayed at the indicated time points using a CellTiter‐Glo 2.0 Cell Viability Assay (Promega, Madison, USA, Cat #G9242), according to the manufacturer's instructions.

The A549 human lung adenocarcinoma cell line (RRID:CVCL_0023) was grown in DMEM (Thermo Fisher Scientific, Waltham, USA, Cat #11960044) supplemented with 10% (v/v) heat‐inactivated FBS and 2% (v/v) penicillin/streptomycin (Thermo Fisher Scientific, Waltham, USA, Cat #15070063) and seeded into 96‐well plates. The following day, triplicate wells of A549 cells were treated with 350 μM paclitaxel from *Taxus ravioli* (Sigma Aldrich, St. Louis, USA, Cat #T7402) or 5 mM H_2_O_2_ (Sigma Aldrich, St. Louis, USA, Cat #H1009) in combination with LAT8881 (0.01–100 μM) or DMSO vehicle alone for 16 h. Cell viability was assayed using a CellTiter‐Glo Cell Viability Assay (Promega, Madison, USA, Cat #G7571), according to the manufacturer's instructions. In some experiments, A549 cells were transfected with 100 nM siRNA specific for human *LANCL1* (Horizon Discovery, Waterbeach, UK, Cat #L‐012166‐00‐0005), *LANCL2* (Origene, Rockville, USA, Cat #SR324535) or control siRNA (Origene, Rockville, USA, Cat #4457289) using Lipofectomine RNAiMAX (Thermo Fisher Scientific, Waltham, USA, Cat #13778100) 16 h prior to paclitaxel/LAT8881 treatment.

### Influenza virus

The IAV strain used in this study was HKx31 (H3N2), which is a high‐yielding reassortant of A/PR/8/34 (PR8; H1N1) that carries the surface hemagglutinin (HA) and neuraminidase (NA) glycoproteins of A/Aichi/2/1968 (H3N2). HKx31 was grown in 10‐day embryonated chicken eggs by standard procedures and titrated on Madin–Darby Canine Kidney (MDCK) cells (RRID:CVCL_0422).

### Influenza virus infection of mice

C57BL/6J male and female mice (6–8 weeks of age) were maintained in the Specific Pathogen‐free Physical Containment Level 2 (PC2) Animal Research Facility at the Monash Medical Centre. All experimental procedures were approved by the Hudson Animal Ethics Committee and experimental procedures carried out in accordance with approved guidelines. For virus infection studies, C57BL/6J mice were randomised. Mice were lightly anaesthetised with isoflurane and intranasally inoculated with 10^4^ pfu of HKx31 (H3N2) IAV in 50 μL PBS, which induces severe disease.^29^, ^49^ Mice were treated at the time points indicated with LAT8881, LAT9991F or LAT7771 (5, 10 or 20 mg kg^−1^; as indicated) in 25 μL PBS via the intranasal route. Control mice were treated with an equivalent volume of PBS alone. Mice were weighed daily and assessed for visual signs of clinical disease, including inactivity, ruffled fur, laboured breathing and huddling behaviour. Animals that lost 20% of their original body weight or displayed severe clinical signs of disease (reduced mobility and rapid breathing) were euthanised. At the indicated time points, mice were sacrificed via intraperitoneal injection of sodium pentobarbital and BAL immediately performed by flushing the lungs three times with 1 mL of PBS. Lungs were then removed and frozen immediately in liquid nitrogen. Titres of infectious virus in lung homogenates were determined by standard plaque assay on MDCK cells (RRID:CVCL_0422).

### Quantification of cytokines in mouse BAL fluid and sera

To detect cytokines, BAL fluid was isolated following centrifugation, and serum was collected and stored at −80°C. Levels of IL‐6, MCP‐1, IFNγ, IL‐10, IL‐12p70 and TNF proteins were determined by cytokine bead array (CBA) using the mouse inflammation kit (BD Biosciences, San Jose, USA, Cat #552364, RRID:AB_2868960).

### Flow cytometry on BAL cells

Cells in the BAL fluid were isolated by centrifugation and treated with red blood cell lysis buffer (Sigma Aldrich, St. Louis, USA, Cat #R7757) for 5 min. The reaction was quenched by washing the cells in FACS buffer (PBS containing 2% (v/v) FBS and 2 mM EDTA). Bronchoalveolar lavage cells were then incubated with fluorescently labelled antibodies at 4°C for 20 min in the presence of Fc receptor blocking monoclonal antibody against CD16/CD32 (clone 93, Thermo Fisher Scientific, Waltham, USA, Cat #139311, RRID:AB_468898) to limit nonspecific antibody binding. Bronchoalveolar lavage cells were stained in FACS buffer with monoclonal antibodies to Siglec‐F (clone E50‐2440, BD Biosciences, San Jose, USA, Cat #565527, RRID:AB_2732831), NK1.1 (clone PK136, BioLegend, San Diego, USA, Cat #108727, RRID:AB_2132706), CD3ε (clone 145‐2C11, BioLegend, San Diego, USA, Cat #100355, RRID:AB_2565969), CD11c (clone HL3, BD Biosciences, San Jose, USA, Cat #564080, RRID:AB_2738580), CD64 (clone X54‐5/7.1, BioLegend, San Diego, USA, Cat #139311, RRID:AB_2563846), Ly6C (clone AL‐21, BD Biosciences, San Jose, USA, Cat #562727, RRID:AB_2737748), Ly6G (clone 1A8, Cat #551461, RRID:AB_394208, BD Biosciences, San Jose, USA), and I‐A^b^ (clone AF6‐120.1, BD Biosciences, San Jose, USA, Cat #562823, RRID:AB_2737818) and the Zombie Aqua viability dye (Cat #423102; BioLegend, San Diego, USA). Total live cells (Zombie Aqua viability dye^−^), neutrophils (Ly6G^+^ Ly6C^int^), NK cells (NK1.1^+^ CD3^−^), T cells (NK1.1^−^ CD3^+^), IM (Ly6G^−^ Ly6C^+^), AM (CD11c^+^ Siglec‐F^+^) and DCs (CD11c^+^ I‐A^b+^) were quantified by flow cytometry using a BD LSRFortessa™ X‐20 (BD Biosciences, San Jose, USA, RRID:SCR_019600) or Aurora flow cytometer (Cytek Biosciences, Fremont, USA, RRID:SCR_019826) and FlowJo™ 10 analysis software (BD Biosciences, San Jose, USA, RRID:SCR_008520). Cells were enumerated using a standard amount of blank calibration particles (ProSciTech, Kirwan, Australia, Cat #QBCP‐60‐5) as determined using a haemocytometer.

For flow cytometric analysis of AM death, BAL cells were treated with Fc receptor blocking monoclonal antibody against CD16/CD32 (clone 93, Thermo Fisher Scientific, Waltham, USA, Cat #139311, RRID:AB_468898) to limit nonspecific antibody binding, followed by staining with fluorochrome‐conjugated monoclonal antibodies (BD Biosciences, San Jose, USA) to Siglec‐F (clone E50‐2440, Cat #565527, RRID:AB_2732831) and CD11c (clone HL3, Cat #553801, RRID:AB_396683). Cells were then incubated with Annexin V (Cat #640912; BioLegend, San Diego, USA) in binding buffer (10 mM HEPES pH 7.4, 150 mM NaCl, and 2.5 mM CaCl_2_) and 5 μg mL^−1^ propidium iodide (PI; Thermo Fisher Scientific, Waltham, USA; Cat #P1304MP). Cells were analysed using a BD FACS Canto II flow cytometer (BD Biosciences, San Jose, USA, RRID:SCR_018056) and FlowJo software (BD Biosciences, San Jose, USA, RRID:SCR_008520).

### Assessment of lung damage

Levels of LDH in BAL fluid supernatant were determined using a CytoTox 96 Non‐radioactive Cytotoxicity Assay (Cat #G1780; Promega, Madison, USA), according to the manufacturer's instructions. Levels of ATP in BAL fluid supernatant were determined by using a CellTiter‐Glo 2.0 Cell Viability Assay (Cat #G9242; Promega, Madison, USA), according to the manufacturer's instructions. Levels of S100A10 in BAL fluid were determined using ELISA (precoated 96 well format; Cat #SEC046Mu‐96 T, Cloud‐Clone Corporation, Katy, USA), according to the manufacturer's instructions.

In the indicated experiments, mice were sacrificed via intraperitoneal injection of sodium pentobarbital, and lungs were immediately inflated and fixed in 10% formalin for at least 24 h, and then processed in paraffin wax. Longitudinal tissue sections (4 μm) were prepared and stained with haematoxylin and eosin (H&E). Tissues were graded for alveolitis and peribronchial inflammation on a subjective scale of 0–5 (0 = no inflammation, 1 = very mild, 2 = mild, 3 = moderate, 4 = marked and 5 = severe inflammation). Sections were also scored for features of epithelial damage such as presence of debris in the airspace, epithelial denudation and thickening of the epithelial wall (0 = no obvious damage, 1 = mild, 2 = moderate, 3 = marked and 4 = severe). Sections were blinded and randomised, and samples corresponding to the least severe and most severe were assigned scores of 0 and 4/5, respectively. All other samples were graded in five random fields by three independent readers. Lung sections were viewed on an Olympus BX60 microscope and photographed at ×10 magnification with a Olympus DP74 colour camera running from Olympus cellSens Dimension software.

### *In vitro* IAV infection

Human primary bronchial epithelial cells (PBECs) were obtained via bronchial lavage as previously described,^50^ from normal subjects who were non‐smokers or had not smoked for > 15 years and had not been diagnosed with asthma or COPD (normal FEV_1_ measurements). Studies were approved by the Monash Health and Monash Medical Centre Human Research Ethics Committee. Consent was obtained from all subjects, and studies were conducted in accordance with the approved guidelines. Human primary bronchial epithelial cells were cultured under submerged conditions on collagen‐coated flasks (Cat #A1064401; Thermo Fisher Scientific, Waltham, USA) in supplemented bronchial epithelial growth medium (BEGM; Cat #CC‐3170; Lonza, Basel, Switzerland) and were used within four passages. Human primary bronchial epithelial cells were plated into collagen‐coated 24‐well plates in BEGM medium without hydrocortisone. The following day, cell monolayers were infected with HKx31 IAV in BEGM media for 1 h at a multiplicity of infection (MOI) of 3. Cell monolayers were then washed and incubated with 100 μM LAT8881 or an equivalent volume of DMSO vehicle alone. Levels of infectious virus in primary cell culture supernatants were determined at 24 h by standard plaque assay on MDCK cells (RRID:CVCL_0422).

### Data and statistical analysis

Data were tested for normality and analysed by GraphPad Prism version 9 software (Graphstats Technologies, RRID:SCR_002798). When comparing three or more sets of values, a one‐way analysis of variance (ANOVA) was used with either a Tukey's or a Dunnett's (when comparisons were only made to IAV‐infected PBS control group) multiple comparisons post hoc test. A Student's *t*‐test was used when comparing two values (two‐tailed, two‐sample equal variance). Survival proportions were compared using the Mantel–Cox log‐rank test. A *P‐*value < 0.05 was considered statistically significant.

## Author contributions

**Christopher M Harpur:** Conceptualization; data curation; formal analysis; investigation; methodology; validation; visualization; writing – original draft; writing – review and editing. **Alison C West:** Conceptualization; data curation; formal analysis; investigation; methodology; validation; visualization; writing – original draft; writing – review and editing. **Mélanie A Le Page:** Data curation; formal analysis; methodology. **Maggie Lam:** Data curation; formal analysis; methodology. **Christopher Hodges:** Data curation; investigation. **Osezua Oseghale:** Data curation. **Andrew J Gearing:** Conceptualization; methodology; project administration; resources; supervision; writing – review and editing. **Michelle D Tate:** Conceptualization; data curation; formal analysis; funding acquisition; investigation; methodology; project administration; resources; supervision; validation; visualization; writing – original draft; writing – review and editing.

## Conflict of interest

The authors of this manuscript have several competing interests. MDT received research funding from Lateral Pharma Pty Ltd. AJG receives consultancy fees from Lateral Pharma Pty Ltd and has patent ownership.

## Supporting information


Supplementary Figure 1
Supplementary Figure 2
Supplementary Figure 3
Supplementary Figure 4
Supplementary Figure 5
Supplementary Figure 6

## Data Availability

The data that support the findings of this study are available from the corresponding author upon reasonable request.
