# Supplementary material for: Naturally derived cytokine peptides limit virus replication and severe disease during influenza A virus infection
Source: Clin Transl Immunology. 2023 Mar 23;12(3):e1443. doi: 10.1002/cti2.1443 (PMC10034483; doi:10.1002/cti2.1443)
Supplement: Supplementary file 1 — Supplementary Figure 1 Supplementary Figure 2 Supplementary Figure 3 Supplementary Figure 4 Supplementary Figure 5 Supplementary Figure 6 [file CTI2-12-e1443-s001.pdf]

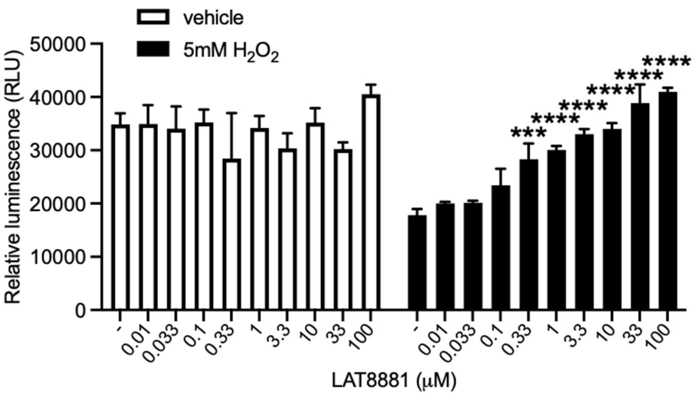

Supplementary figure 1. LAT8881 improves cell survival in human cells. Viability of human A549 cells 16 h following treatment with 5 mM H_2O_2 ± LAT8881 (0.01-100 μM), as determined by luminescent ATP detection ± SD. ****P* < 0.001, *****P* < 0.0001 vs H_2O_2 alone, two-way ANOVA. Data are representative of two independent experiments.

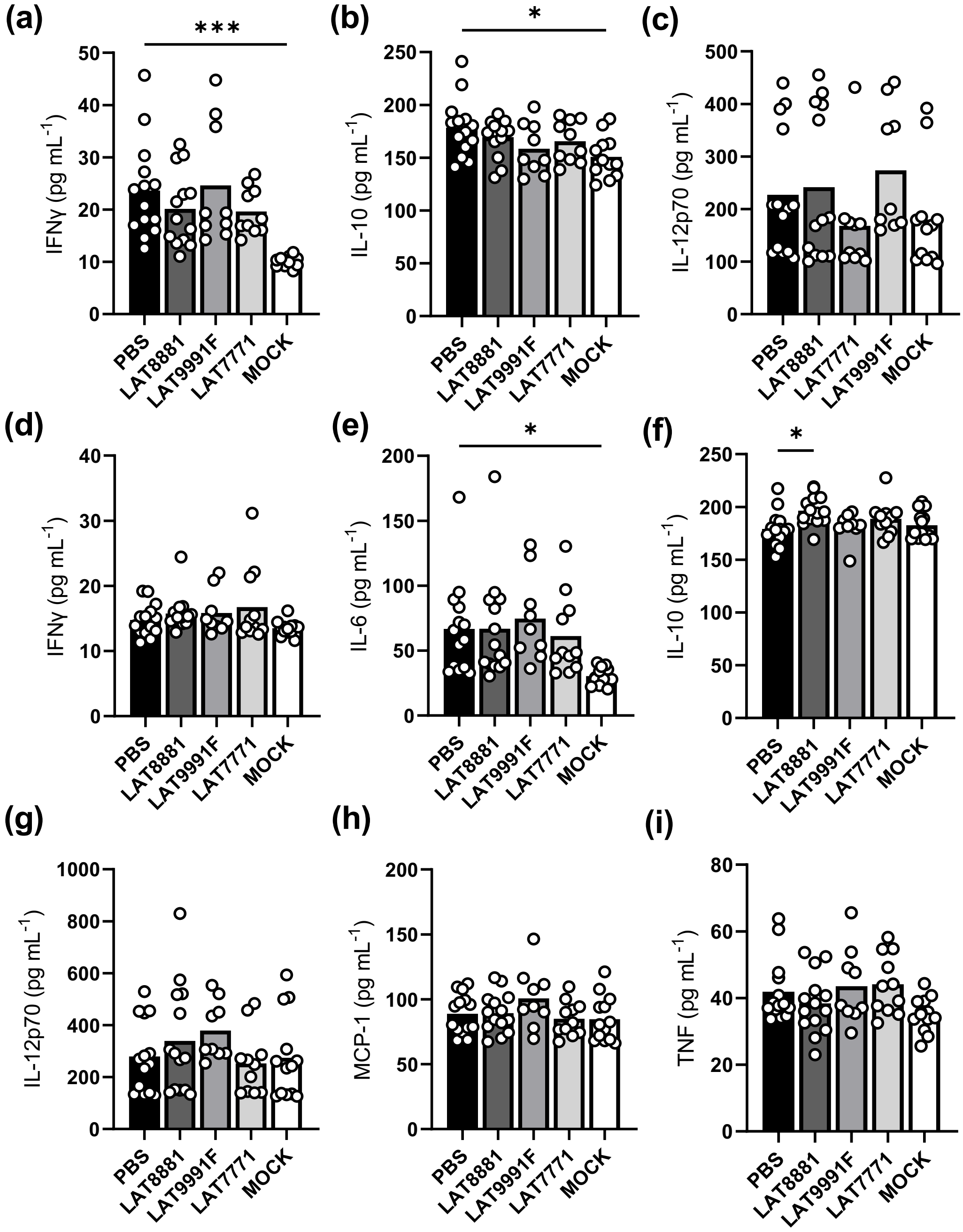

Supplementary figure 2. LAT8881, LAT9991F or LAT7771 treatment during severe IAV infection has negligible effects on IFNγ, IL-10 and IL-12p70 levels in BAL fluid or on any assessed cytokine in serum. Groups of male C57BL/6 mice received daily i.n. treatment with 20 mg kg^{-1} of LAT8881, LAT9991F or LAT7771 from 1 dpi with 10^4 pfu of HKx31 IAV and BAL fluid and blood serum were collected at 3 dpi. MOCK-infected and IAV-infected control mice received PBS alone. BAL fluid concentration of IFNγ **(a)**, IL-10 **(b)** and IL-12p70 **(c)** determined by cytokine bead array. Blood serum levels of IFNγ **(d)**, IL-6 **(e)**, IL-10 **(f)**, IL-12p70 **(g)**, MCP-1 **(h)** and TNF **(i)** determined by cytokine bead array. Data are presented as the mean with each data point representing an individual animal. *n* = 9-14, pooled from at least two independent experiments. **P* < 0.05, ****P* < 0.001, one-way ANOVA with Dunnett's multiple comparisons test.

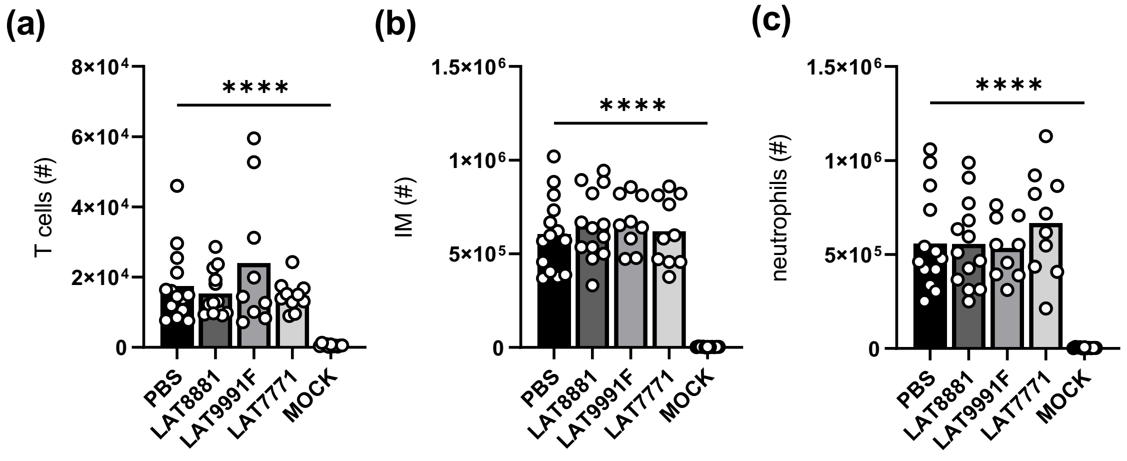

Supplementary figure 3. Comparable numbers of T cells, inflammatory macrophages and neutrophils in the BAL fluid of IAV-infected mice following LAT8881, LAT9991F or LAT7771 treatment. Groups of male C57BL/6 mice received daily i.n. treatment with 20 mg kg^{-1} of LAT8881, LAT9991F or LAT7771 from 1 dpi with 10^4 pfu of HKx31 IAV and BAL fluid was collected at 3 dpi. MOCK-infected and IAV-infected control mice received PBS alone. Numbers (#) of T cells **(a)**, inflammatory macrophages (IM), **(b)** and neutrophils **(c)** in BAL fluid determined by flow cytometry. Data are presented as the mean with each data point representing an individual animal. n = 9-14, pooled from at least two independent experiments. ****$P < 0.0001$, one-way ANOVA with Dunnett's multiple comparisons test.

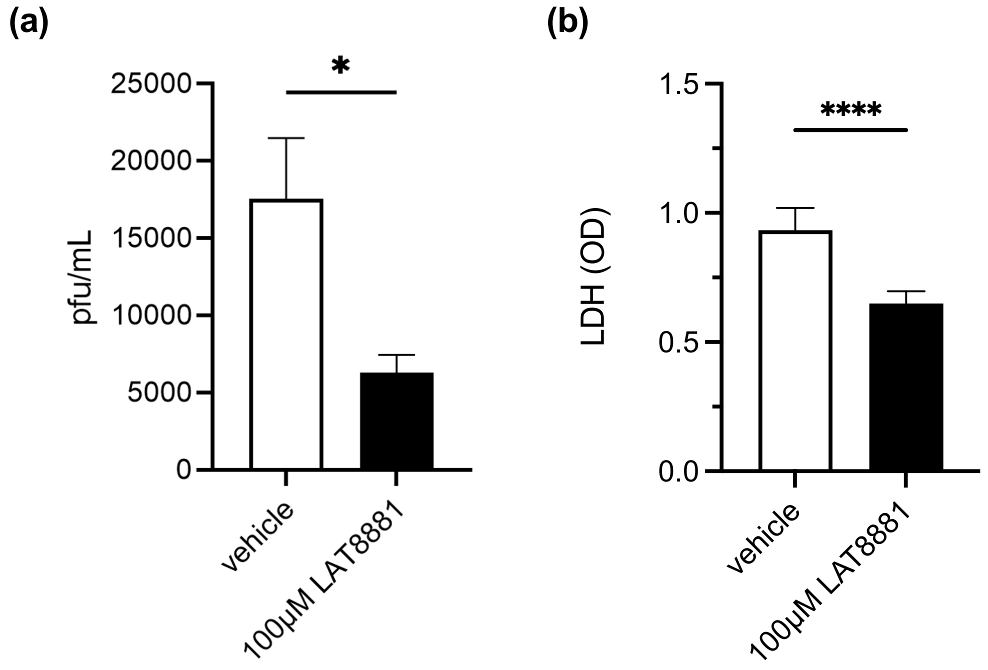

Supplementary figure 4. LAT8881 limits IAV replication in human primary bronchial epithelial cells. Human primary bronchial epithelial cells (PBECs) from 3 patients were treated with 100 µM of LAT8881 or vehicle alone, 1 h following infection with HKx31 IAV infection (multiplicity of infection of 3). **(a)** Levels of infectious virus in cell supernatants were determined at 24 h post-inoculation by standard plaque assay on MDCK cells. **(b)** LDH levels in PBEC culture supernatants were used to assess cell viability determined by colorimetric (OD; optical density) assay. Data are presented as mean ± SD and pooled from n = 3 (a) and n = 2 (b) patients using experimental triplicates. *$P < 0.05$, ****$P < 0.0001$, Student's t-test. Data are representative of three independent experiments.

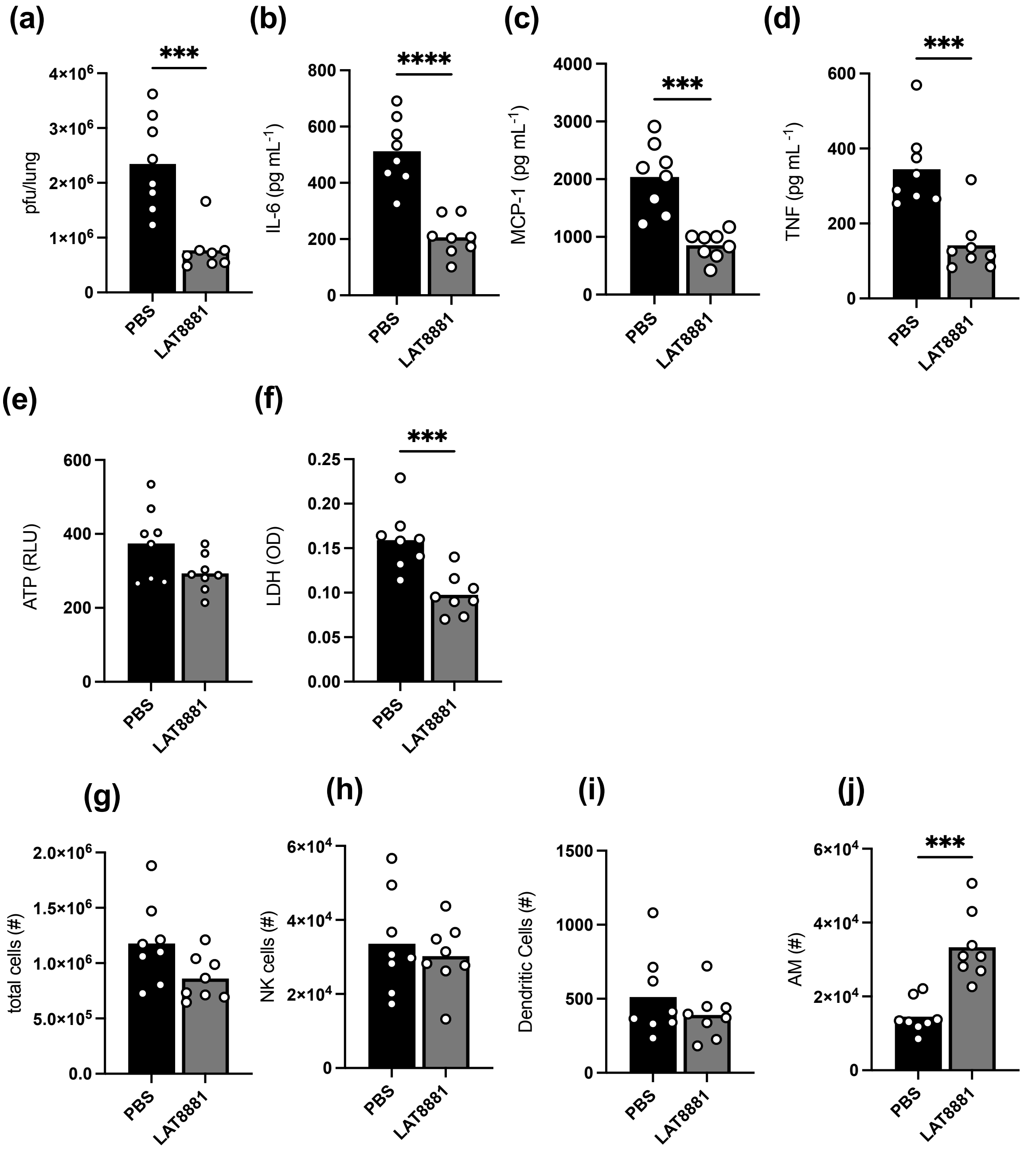

Supplementary figure 5. LAT8881 treatment during severe IAV infection has comparable efficacy in female mice. Groups of female C57BL/6 mice received daily i.n. treatment with 20 mg kg^{-1} of LAT8881 from 1 dpi with 10^4 pfu of HKx31 IAV. BAL fluid and lung tissues were collected at 3 dpi. IAV-infected control mice received PBS alone. **(a)** Lung viral loads (pfu/lung) measured by a standard plaque assay. BAL fluid concentration of IL-6 **(b)**, MCP-1 **(c)** and TNF **(d)** determined by cytokine bead array. Levels of ATP **(e)** and LDH **(f)** in BAL fluid determined by luminescent (RLU; raw luminescence units), colorimetric (OD; optical density) assays or ELISA, respectively. Numbers (#) of total viable cells **(g)**, NK cells **(h)**, dendritic cells **(i)** and AM **(j)** in the BAL, as determined by flow cytometry. Data are presented as the mean with each data point representing an individual animal. n = 8. *P < 0.05, **P < 0.01, ***P < 0.001, ****P < 0.0001, Student's t-test.

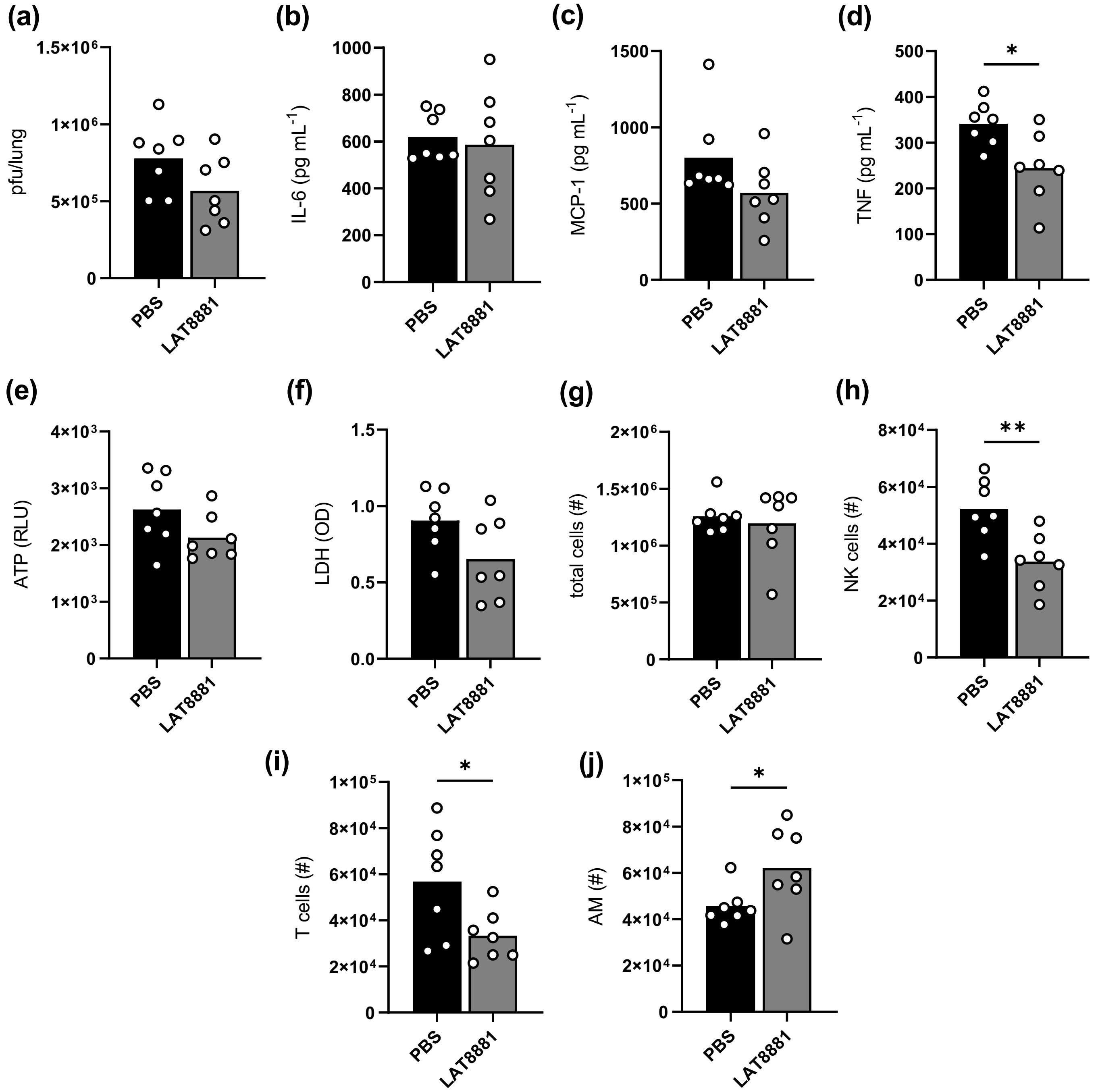

Supplementary figure 6. LAT8881 treatment at the peak of IAV disease. Groups of C57BL/6 mice received daily i.n. treatment with 20 mg kg^{-1} of LAT8881 from 3 dpi with 10^4 pfu of HKx31 IAV. BAL fluid and lung tissues were collected at 5 dpi. IAV-infected control mice received PBS alone. **(a)** Lung viral loads (pfu/lung) measured by a standard plaque assay. BAL fluid concentration of IL-6 **(b)**, MCP-1 **(c)** and TNF **(d)** determined by cytokine bead array. Levels of ATP **(e)** and LDH **(f)** in BAL fluid determined by luminescent (RLU; raw luminescence units), colorimetric (OD; optical density) assays or ELISA, respectively. Numbers (#) of total viable cells **(g)**, NK cells **(h)**, T cells **(i)** and AM **(j)** in the BAL, as determined by flow cytometry. Data are presented as the mean with each data point representing an individual animal. n = 7. *P < 0.05, **P < 0.01, Student's t-test.
